# A Multi-Subject Tractography Atlas of Human Cerebellar Connections

**DOI:** 10.21203/rs.3.rs-9742595/v1

**Published:** 2026-06-01

**Authors:** Daniela Carrasco Guerrero, Natalia Vidal Barraza, Patricio Riquelme, María E. Castelló, Joseph Yuan-Mou Yang, Cecilia Hernández, Pamela Guevara

**Affiliations:** 1 Electrical Engineering Department, Faculty of Engineering, Universidad de Concepción, Concepción, Chile.; 2 Faculty of Engineering, Universidad de Concepción, Concepción, Chile.; 3 Department of Medical Technology, Faculty of Medicine, Universidad de Chile, Santiago, Chile.; 4 Laboratory of Neuropsychology and Clinical Neurosciences, Faculty of Medicine, Universidad de Chile, Santiago, Chile.; 5 Desarrollo y Evolución Neural, Depto. Neurociencias Integrativas y Computacionales, Instituto de Investigaciones Biológicas Clemente Estable, Montevideo, Uruguay.; 6 Neuroscience Advanced Clinical Imaging Service (NACIS), Department of Neurosurgery, The Royal Children’s Hospital, Melbourne, Australia.; 7 Neuroscience Research, Murdoch Children’s Research Institute, Parkville, Melbourne, Australia.; 8 Department of Paediatrics, University of Melbourne, Parkville, Melbourne, Australia.; 9 Computer Science Department, Faculty of Engineering, Universidad de Concepción, Concepción, Chile.

**Keywords:** cerebellum, white matter atlas, diffusion MRI, tractography, cerebellar pathways, fiber bundle segmentation

## Abstract

The cerebellum is a highly interconnected structure involved in motor coordination and balance as well as cognitive, sensory, autonomic, and emotional functions. Its connectivity relies on complex afferent and efferent white matter pathways linking it with the brainstem, spinal cord, thalamus, cerebral cortex, vestibular system, and limbic-related structures. Recent advances in diffusion MRI (dMRI) and tractography have enabled the in vivo reconstruction of these pathways, offering new opportunities to study cerebellar anatomy and connectivity. However, compared with supratentorial white matter, cerebellar connections remain underexplored owing to their smaller size, anatomical complexity, and the absence of comprehensive tractography atlases.

We propose a white matter fiber atlas of human cerebellar connections based on regions of interest (ROIs) derived from 30 young healthy subjects from the Human Connectome Project (HCP). A total of 23 ROIs were segmented to reconstruct, via tractography, 20 cerebellum-associated pathways, including 8 bilateral pathways and 4 interhemispheric connections. These comprise the three cerebellar peduncles and the cerebello-ponto-hypothalamic, parieto-ponto-cerebellar, fronto-ponto-cerebellar, occipito-ponto-cerebellar, contralateral cerebello-thalamo-cortical, dentato-rubro-thalamo-cortical, olivocerebellar, dorsal spinocerebellar, and periaqueductal gray–cerebellar pathways. A neuroanatomical expert evaluated the 20 atlas tracts using four qualitative criteria, finding good anatomical fidelity with an overall mean score of 3.9 out of 5. The same reconstruction procedure was then applied to 25 additional HCP subjects to evaluate the automatic segmentation of these bundles in unseen subjects. The proposed atlas provides a comprehensive framework for studying cerebellar structural connectivity and its relationship with motor and non-motor functions. It enables quantitative tractometry analyses derived from diffusion MRI models and facilitates the integration of multimodal and functional information. This atlas may support future research in neuroscience, neuropsychiatric disorders, and neurosurgical planning by providing a standardized representation of cerebellar white matter pathways.

## 1 Introduction

The cerebellum is a quasi-crystalline structure present in all vertebrates except hagfish [1]. In addition to its well-known role in motor functions such as balance maintenance, movement coordination, muscle memory, and sensorimotor integration [2–5], it serves as an interface between sensorimotor function and cognition, including the sense of self [6, 7]. Recent research has revealed its involvement in higher cognitive and autonomic functions [2, 8], including attention, language control, and blood pressure regulation [9, 10]. This expanded understanding of cerebellar function has prompted renewed interest in studying its structure and connectivity, particularly the direct connections between the hypothalamus and the cerebellum that have recently been identified [11]. Recently, efferent pathways belonging to non-motor functions such as social behavior, control of fear, memory, reward, and general behavioral functions were demonstrated [12]. This mostly involves an anatomic structure located in the midbrain called the ventral tegmental area (VTA), a major source of dopamine neurons, as well as the dentate and fastigial nuclei [13].

The cerebellum is connected to the brainstem and the brain, forming the encephalon. Its main connection pathways to the aforementioned structures are the three cerebellar peduncles: superior cerebellar peduncle (SCP), middle cerebellar peduncle (MCP) and inferior cerebellar peduncle (ICP). These pathways are responsible for transmitting information bidirectionally between the cerebellum and the rest of the central nervous system, facilitating the proper functioning of human motor, cognitive, sensory, and autonomic activity [14].

This cerebellar-loop system is organized as a multichannel network of parallel pathways that keep signals separate throughout the circuit, enabling distinct input and output routes. The cerebro-cerebellar loop includes inputs from the frontal, premotor, and motor cortices, with these fibers converging at the pontine nuclei and reaching the cerebellum through the contralateral MCP. Another important pathway begins from the dentate nuclei, ascending directly to the contralateral red nucleus within the ventral tegmental area (dentato-rubro-thalamic tract) [15]. The spino-cerebellar loop is involved with the somatosensory cortex, visual and auditory receptors, and the spinal cord through the dorsal and ventral spino-cerebellar tracts [16]. Additionally, the vestibulo-cerebellar loop integrates balance and gaze control information, connecting the vestibular system with the flocculonodular lobe [13].

Histoarchitecturally, the cerebellum consists of a foliated superficial cortex surrounding deep cerebellar nuclei. In humans, it is composed of about 86 billion neurons, representing 80% of the total number of encephalic neurons, suggesting an astounding computational capacity [1]. There are considerable differences in the gross morphology of the cerebellum and the cerebellum-like structures, in sizes and shapes, across vertebrates, with the cerebellum being the most variable central nervous system structure, reaching about 60% of total encephalon volume in both sharks [7] and mormyrids [4].

The cerebellar cortex is characterized by a modular organization already depicted by Cajal [17]. The basic cerebellar circuit comprises the following elements: i) Purkinje neurons, the central units of the cerebellar cortex circuit whose soma form a monolayer that divides the cerebellar cortex into the superficial molecular layer, where their fan-shaped dendrites extend profusely, and the deep granular layer traversed by Purkinje neuron’s axons on their way to the deep cerebellar nuclei: ii) Climbing fibers that branch at the cerebellar molecular layer, mirroring the Purkinje cell’s apical dendrite tree on which they establish excitatory synapses. They originate mainly from the inferior olivary nucleus, although other structures that originated them are unknown. The information carried by these fibers comes from different areas, as the inferior olivary nucleus receives afferents from the proprioceptive organs, the red nucleus, and the periaqueductal gray and is therefore involved in multiple functions and structures [18]; iii) Granule cells, whose soma populate the granular cell layer and whose axons project to the molecular layer and contact the apical dendrites of Purkinje cells; iv) Mossy fibers, whose end terminals contact granular cell dendrites and axon terminals of Golgi cells. Most mossy fibers convey information from the vestibular system or the reticular formation nuclei, while others use the connection between the cerebral hemispheres and the cerebellum to deliver information from the cerebral cortex indirectly through pontine connections. These fibers transmit sensory information mainly from the muscles, joints, and skin. Within the cerebellar cortex, mossy fibers provide excitatory input to granule cells, whose axons form parallel fibers that modulate the dendritic trees of Purkinje cells [19].

Studies on cerebellar connectivity first started with post-mortem dissection of animal models to analyze fiber orientation [11]. Advances in methodological approaches for studying brain structure in animal models, including histological tract-tracing, degeneration techniques, and anterograde, retrograde, and transneuronal tracing methods, significantly deepened the understanding of the cerebellum’s white matter organization, particularly its connections with the cerebrum. These techniques uncovered the underlying circuit architecture of cerebro-cerebellar networks, revealing functionally distinct neuronal subpopulations linked by specific white matter pathways [15].

More recently, diffusion magnetic resonance imaging (dMRI) [20] made cerebellar connectivity analysis possible. Using software tools like DSI Studio [21], it is possible to process these images through models such as Diffusion Tensor Imaging (DTI) [22] and Generalized Q-Sampling Imaging (GQI) [23], which allow for the reconstruction of the desired fibers through deterministic tractography [24]. Tractography has allowed the observation of the tracts of the brain using dMRI, thus enabling us to expand our knowledge of the brain’s deep connections and structures through more direct analysis, especially in complex and inaccessible regions such as the cerebellum. Over the last two decades, these methods have led to the development of numerous atlases of deep white matter bundles [25–27] and, more recently, superficial white matter bundles [26, 28]. However, cerebellar connections remain comparatively underexplored because of their smaller size and complex anatomy [29].

Besides its usefulness for studying cerebellar connections under different conditions, the importance of tractography in the medical field lies in its application for planning surgical approaches when the cerebellum is compromised by a tumor or injury. Since cerebellar connections are difficult to delineate with conventional techniques, tractography can guide safer and more precise interventions [30]. Likewise, its usefulness has been proposed for studying the relationship between the cerebellum and neuropsychiatric disorders, in that way enabling the development of better solutions and increasing the accuracy of these diagnoses [31].

Although several cerebellar atlases have been proposed, most focus on specific regions or probabilistic gray matter maps, such as the probabilistic atlases of the cerebellar lobes [32, 33]. In contrast, anatomical or white matter (WM) fiber atlases enable faster and more standardized segmentation than traditional manual or structure-specific approaches, thereby facilitating comparative studies between clinical populations and healthy controls. However, a significant gap remains in the standardized representation of cerebellar white matter pathways, as current atlases largely overlook the continuous fiber trajectories that connect and integrate cerebellar regions.

In terms of white matter tractography, Lyu et al. created a multimodal submillimeter MRI atlas of the human cerebellum based on a young caucasian male [34]. The tractography data were calculated using asymmetric fiber orientation distribution functions (AFODF) [35] and active cortex tractography (ACT) [36]. The method used a whole-brain anatomical parcellation and voxel annotation based on fiber clusters, followed by a refinement to obtain a final bundle segmentation. The atlas included 13 bundles to the frontal cortex, 6 bundles to the motor cortex, 3 bundles to the parietal cortex, 3 bundles to the occipital cortex, 3 bundles to the occipital and temporal cortices, 12 bundles to the subcortical structures, and 9 bundles to the pons and medulla. Another work related to cerebellar connectivity created a probabilistic atlas (in NIFTI format) of human brainstem pathways based on 1300 ROIs manually delineated on 20 HCP subjects [37]. It is composed of 23 brainstem pathways, including the three cerebellar peduncles (SCP, MCP, and ICP). More specifically, the SCP is mainly composed of the anterior spinocerebellar tract, the cerebellorubral tract, and the cerebellothalamic tract. The ICP mainly consists of afferent tracts from the medulla oblongata to the cerebellum and the vestibulocerebellar tract.

For these reasons, this study proposes the creation of a fiber atlas of human cerebellar connections based on regions of interest (ROIs) derived from 30 healthy subjects from the Human Connectome Project (HCP) [38]. For its construction, 23 regions of interest were segmented to generate a total of 20 cerebellum-associated pathways, including 8 bilateral connections and 4 connections spanning both hemispheres. These include the three cerebellar peduncles, and the cerebello-ponto-hypothalamic, parieto-ponto-cerebellar, fronto-ponto-cerebellar, occipito-ponto-cerebellar, contralateral cerebello-thalamo-cortical, dentato-rubro-thalamo-cortical, olivocerebellar, dorsal spinocerebellar, and periaqueductal gray cerebellar pathways. Additionally, we used the same procedure to reconstruct these cerebellar connections of another 25 subjects from the HCP database in order to evaluate the automatic segmentation of these fiber bundles in new subjects.

To the best of our knowledge, this is the first multi-subject atlas of cerebellar white matter connections based on dMRI tractography containing such a high range of cerebellar pathways. Although the proposed atlas presents limitations and opportunities for improvement, it represents an important step toward the development of comprehensive tools for advancing our understanding of cerebellar structure and function. Furthermore, it may facilitate the identification of structural patterns and alterations associated with neurological disorders through quantitative tractometry metrics derived from diffusion MRI models, while also enabling the integration of functional and multimodal information.

## 2 Materials and Methods

### 2.1 Neuroimaging data

Diffusion and structural MRI data from 55 healthy young adult subjects were obtained from the Human Connectome Project (HCP) 1200 database [38]. Data from thirty subjects were used for atlas construction, and data from another group of 25 subjects were used for automatic segmentation evaluation. All data were acquired on a customized Siemens 3T Connectom scanner [39] using a multi-shell dMRI protocol consisting of three b-values (1000, 2000, and 3000 s/mm^2^) with 90 diffusion-encoding directions per shell, yielding a total of 270 diffusion-weighted volumes plus 18 interspersed b=0 volumes, at an isotropic voxel size of 1.25 mm. The HCP minimally preprocessed data were used [40], which include corrections for gradient nonlinearity, head motion, eddy current-induced distortions, and susceptibility distortions [41, 42], with diffusion data aligned to the native structural space. Subject-specific nonlinear transformation matrices from native structural space to MNI152 space, computed using FNIRT [43], were also provided. All subjects provided written informed consent under protocols approved by the relevant Institutional Review Boards, as described in [44].

### 2.2 Imaging data analysis

#### 2.2.1 Definition of pathways and anatomic structures

First, we conducted a literature search using anatomical reviews and studies of specific cerebellar connections to define cerebellar and extracerebellar structures and their associated pathways. The selection criteria considered the relevance and functional role of each structure. The collected information included the specific cerebellar and cerebral regions involved, the pathways and fiber trajectories, and whether each pathway was afferent or efferent, despite the fact that tractography cannot determine the directionality of these connections. We identified connections such as ponto-cerebellar pathways [31, 45], cortico-pontine projections [46], and cerebellar circuits [47].

Although numerous tracts involve the cerebellum, this study focused on the largest and best-characterized pathways described in the literature, particularly those associated with cognitive and motor functions [48, 49]. The cerebellum contributes to cognitive brain networks through complex cerebro-cerebellar loops that are essential for higher-order neural processes, including attention, executive control, and working memory [50]. Despite significant advances in understanding these mechanisms, the precise nature of the cerebellum’s contribution to cognition remains incompletely understood [51]. Within the cerebellum lie the most important pathways, specifically the superior (SCP), middle (MCP), and inferior (ICP) cerebellar peduncles. These structures act as the primary anatomical gateways for the cerebro-cerebellar loops, facilitating the bidirectional flow of information necessary for both motor coordination and complex cognitive processing [50].

In total, 20 connections were defined, including 8 bilateral connections and 4 connections spanning both hemispheres, as shown in Table 1. The only bilaterally represented tracts without hemispheric subdivision are the middle cerebellar peduncle (MCP), the olivocerebellar tract (OVC), the periaqueductal gray cerebellar connections (PAG), and the superior cerebellar peduncle (SCP).

The selection of these connections is further justified by their increasing clinical relevance. Recent neuroimaging studies have demonstrated that disruptions in these specific white matter tracts are hallmark features of various neurological and neurodevelopmental conditions. For instance, aberrant connectivity within the cerebello-thalamo-cortical (CTC) circuit has been linked to the pathophysiology of motor tremors and gait instability [52]. Similarly, a correlation has been found between patterns of atrophy in the dentato-rubro-thalamo-cortical tract (DRTC) in patients with medulloblastoma and the severity of symptoms such as mutism, ataxia, and dysmetria [53]. Furthermore, it has also been found that the microstructural integrity of the DRTC tract is affected differently across its segments. Specifically, the region of the fascicle connecting the dentate nucleus and the thalamus was more severely affected in Friedreich’s ataxia than its segments extending from the thalamus to the cortex [54]. In neurodevelopmental disorders such as ADHD, there have been alterations in the white cerebellar matter for projection, commissural, and association pathways of individuals with ADHD, which were associated with symptom severity and cognitive deficits [55, 56].

The detailed description of each tract is provided in the Supplementary File 1, section 1. The tracts were reconstructed based on the definition of regions of interest and deterministic tractography using DSI Studio software [21].

#### 2.2.2 Definition of regions of interest

According to the identified tracts and the anatomical structures involved, the regions of interest (ROIs) required for tract reconstruction were defined. These ROIs are indicated in italics in the description of each tract provided below.

##### Superior cerebellar peduncle (SCP).

The superior cerebellar peduncle is the main connection pathway between the cerebellum and the cerebrum. It is considered both an afferent and an efferent pathway, indicating that it not only receives information from the cerebrum but also transmits cerebellar output. It is primarily involved in cognitive functions and the coordination of muscular activity [57]. The principal structures through which the fibers of the **SCP** pass include the thalamus, dentate nucleus, globose nucleus, emboliform nucleus, and red nucleus.

##### Middle cerebellar peduncle (MCP).

The middle cerebellar peduncle uses the cerebellopontine connections to perform functions such as the planning and programming of voluntary muscle movements. It is considered an afferent pathway that receives information through its connection with the cerebral cortex. The **MCP** is regarded as the cerebellar structure containing the greatest number of fiber projections. The main structures involved in this pathway include the trigeminal nerve, pons, most cerebellar lobules, and the dentate nucleus [57].

##### Inferior cerebellar peduncle (ICP).

The inferior cerebellar peduncle carries both afferent and efferent tracts connecting the vestibular system and the spinal cord. Therefore, it plays an important role in maintaining body awareness of the surrounding environment and contributes to balance control. In addition, it is responsible for transmitting proprioceptive information to the brain. The **ICP** is associated with the dorsolateral medulla, the pontomedullary junction, the contralateral cerebellar cortex, and the fourth ventricle [57].

Rather than representing a single tract, each cerebellar peduncle contains several distinct fiber pathways that serve as the principal communication routes between the cerebellum and the brainstem, spinal cord, and cerebrum. Although the cerebellar peduncles are not individual tracts, they represent the most commonly segmented cerebellar bundles and are frequently included in white matter fiber atlases. For this reason, they were incorporated into our atlas, together with more specific cerebellar connections that course through these peduncles.

##### Fronto-ponto-cerebellar tract (FPC).

This tract involves the frontal lobe, which is part of the premotor cortex [58], passing through the tegmentum and the pons before entering the cerebellum through the SCP and MCP. The FPC tract can be reconstructed using two ROIs: one placed in a cerebellar hemisphere, specifically in the **MCP**, defined on the coronal plane of the dorsal surface of the brainstem, and a second ROI placed in the **contralateral frontal lobe**, defined as anterior to the sulcus on the axial plane, just above the cingulate gyrus [45].

##### Parieto-ponto-cerebellar tract (PPC).

This tract is involved in the somatosensory association cortex. This cortical function allows spatial localization of the objects surrounding us and proprioception [45], which refers to the awareness of the position and orientation of our body and its parts. Similar to the FPC tract, the **MCP** is used as the seed region, since it represents a major connection pathway within the cerebellum, while an additional ROI is placed in the **contralateral parietal lobe** [45].

##### Occipito-ponto-cerebellar tract (OPC).

This tract originates from the **occipital cortex** posterior to the parieto-occipital sulcus, passing through the internal capsule and continuing along the dorsolateral portion of the crus cerebri, surrounded by the corticospinal tract. It then descends through the basis pontis, where it crosses to the opposite side of the pons and traverses the contralateral **MCP** [59].

##### Temporo-ponto-cerebellar tract (TPC).

The three previously described tracts, together with the temporo-ponto-cerebellar tract, belong to the anatomical group of the cerebro-ponto-cerebellar tracts [31]. In particular, this tract arises from the anteroinferior portion of the temporal lobe, ascending dorsally toward the uncinate fasciculus and ventrally toward the lateral ventricle. It then descends along the posterior margin of the crus cerebri, ventrally surrounded by the OPC tract up to the level of the pons, where it is located dorsolaterally to both the OPC and corticospinal tracts. Finally, similarly to the other previously mentioned tracts, it descends contralaterally to the cerebellum through the MCP [59]. This tract was ultimately not included in our atlas due to the difficulty of obtaining a reliable and reproducible segmentation across subjects.

##### Dentato-rubro-thalamo-cortical tract (DRTC).

Traditionally described as a decussating pathway ascending toward the contralateral thalamus, recent studies using deterministic tractography in healthy subjects, as well as brain microdissection techniques, have demonstrated the existence of a non-decussating pathway [15]. This tract originates in the cerebellum, specifically in the **dentate nucleus**, ascending through the brainstem, where most of its axons cross the midline within the SCP to synapse in the contralateral red nucleus of the midbrain. Technically, the DRTC terminates in the **thalamus**; however, for functional completeness, in [27] they extended its trajectory to terminate in the cortex.

##### Dorsal spinocerebellar tracts (SPNC).

The dorsal spinocerebellar tract ascends through the dorsolateral medulla oblongata and terminates in the vermis and paravermis of the anterior lobe. It appears flattened at the level of the restiform corpus, traverses the cerebellum within the **ICP** and outside the SCP in the superior portion of the cerebellum, projecting preferentially to the vermal regions of lobules I-V (afferent pathway) [29]. For segmentation, the seed ROI corresponds to the ICP and the second ROI the **ipsilateral cerebellar hemisphere**.

##### Olivocerebellar tract (OVC).

This pathway consists of axonal projections originating from the **inferior olive** (IO) and terminating in the cerebellar cortex. From their origin, these fibers run medially to cross over at the midsagittal plane, passing either through or above the contralateral inferior olive. They then reach the medullary white matter beneath the lateral surface, eventually integrating into the inferior cerebellar peduncle (ICP) [60, 61]. The trajectory of the OVC has been partially identified using the inferior olivary nucleus as the main ROI, revealing pathways entering the cerebellum through the ICP and connecting both the dentate nucleus and the cerebellar cortex [62].

##### Cerebello-ponto-hypothalamic tract (CPH).

The CPH enters the pons, where it decussates contralaterally through the transverse pontine fibers after crossing to the opposite hemisphere. The tract then ascends through the pons toward the midbrain, continuing to the junction of the cerebral peduncle and the midbrain tegmentum. Subsequently, the CPH projects toward the posterior aspect of the cerebral peduncle and ascends alongside the temporopontine tract. It then deviates laterally toward the medial surface of the parahippocampal gyrus. At the medial margin of the parahippocampal gyrus, the CPH changes direction and projects ventrally along the superior and lateral surfaces of the optic tract. Distally, the CPH projects more medially relative to the optic tract. Continuing its course, the CPH travels ventrally along the anterior columns of the fornix. Finally, the CPH terminates in the anterior region of the **hypothalamic nucleus**, just posterior to the optic chiasm [63].

While the fastigial and interposed nuclei are more closely associated with basic vestibular and motor functions [64], the dentate nucleus serves as the primary output center to associative and autonomic areas of the forebrain. Given that the hypothalamus is a key regulatory center for homeostasis and the limbic system [11], the **ventral dentate nucleus** is used as a seed ROI in this study.

##### Vestibulocerebellar tract (VCT).

The primary VCT originates from the superior and medial vestibular nuclei at the level of the pons, passing through the ICP and terminating in the ipsilateral uvula-nodulus of the cerebellum. The secondary component of the VCT originates from the inferior and medial vestibular nuclei at the level of the medulla oblongata, crossing the cerebellar vermis to the contralateral side and terminating bilaterally in the uvula-nodulus of the cerebellum [65]. This tract plays an important role in the central vestibular system and is involved in the perception of head and body movement in spatial orientation, as well as in posture control [66]. This tract was ultimately not included in our atlas due to the difficulty of obtaining a reliable and reproducible segmentation across subjects.

##### Cerebello-thalamo-cortical tract (CTC).

This pathway originates in the deep cerebellar nuclei, specifically the Dentate Nucleus (DN). Axons from the DN exit the cerebellum via the Superior Cerebellar Peduncle (SCP), which acts as the major outflow tract [67]. Upon reaching the midbrain, the fibers undergo a complete decussation (the decussation of the SCP) before ascending to the contralateral Red Nucleus (RN). From the RN, the pathway continues its rostral trajectory to synapse in the ventrolateral (VL) nucleus of the thalamus, finally projecting to the motor and premotor cerebral cortices [62]. The CTC is an efferent pathway that connects the cerebellum with the cerebrum through the SCP and the contralateral thalamus [68]. Several methods have been used to reconstruct the fibers of the CTC tract. However, difficulties have been reported in reliably reconstructing the contralateral fibers of this pathway, even when a contralateral ROI is selected [58]. For segmentation, we used the **ventral dentate nucleus** as the seed region and the **contralateral red nucleus** as the ROI.

##### Cerebello-periaqueductal gray connection (PAG)

The periaqueductal gray functions as a “control station” for innate and learned responses to stressful stimuli, and its fibers are connected with the fastigial nucleus, interposed nucleus, dentate nucleus, and cerebellar lobules [69]. We used the **periaqueductal gray** as a seed to segment this connection. We used the ROI definitions provided by DSI Studio [21], used to perform the tract reconstructions. In this software, *seeds* correspond to the structures from which tract reconstruction is initiated, whereas *ROIs* are the regions through which the reconstructed pathways must pass, thereby constraining and shaping the trajectories of the tracts [48].

#### 2.2.3 Anatomical ROI Segmentation

Two types of segmentation were applied to the selected ROIs:

##### Automatic segmentation.

Automatic segmentations were performed using the atlases available in DSI Studio. Table 2 summarizes the selected ROIs included in this study. All ROIs were visually inspected to verify their anatomical accuracy and proper alignment.

##### Manual segmentation.

A total of 10 anatomical structures were manually segmented for each subject, as shown in Table 3. These structures included the hypothalamus, inferior olivary nucleus, thalamus, middle cerebellar peduncle, superior cerebellar peduncle, and inferior cerebellar peduncle. To ensure anatomical accuracy and validate the manual approach, all segmentations were based on detailed anatomical knowledge and validated by a medical technologist with extensive expertise in neuroanatomy.

#### 2.2.4 Tract generation

Once all regions of interest (ROIs) had been segmented, deterministic tractography was performed. For each tract, one ROI was used as the seed region, while additional ROIs were defined as regions through which the streamlines pass or terminate. The Generalized Q-sampling Imaging (GQI) reconstruction model [23] was used for diffusion modeling due to its ability to resolve complex fiber configurations and crossing tracts. GQI is a non-parametric, model-free approach that estimates the Spin Distribution Function (SDF), which represents the orientation and density of water molecules along different directions. By identifying multiple local maxima (peaks) within the SDF, GQI can effectively resolve crossing fibers, a common occurrence in regions such as the brainstem and the lateral projections of the cerebellum, where different systems intersect.

For tract reconstruction, deterministic streamline propagation tractography was performed in DSI Studio [74]. Once the ROIs and seed regions were defined, tractography parameters were adaptively adjusted according to each subject and pathway. This individualized approach was implemented to account for inter-subject anatomical variability and localized differences in white matter integrity, thereby facilitating the identification of the most morphologically consistent fiber patterns. The tracking threshold, based on Quantitative Anisotropy (QA), was set between 0.04 and 0.1. Unlike fractional anisotropy, QA serves as a more robust termination criterion in deterministic tractography because it effectively reduces the influence of noise and partial-volume effects by quantifying the density of spins along specific orientations. The angular threshold was set to 0 (default), allowing a randomized range between 45° and 90°, thereby facilitating the reconstruction of high-curvature pathways commonly observed in cerebellar anatomy. Furthermore, the step size was set to 0 (corresponding to a randomized range between 0.5 and 1.5 mm), with the exception of the CPH tract, for which a step size of 0.1 mm was used. Fourth-order Runge–Kutta (RK4) integration was employed to ensure high spatial precision during fiber propagation. Each tract was initially reconstructed with 4,000 streamlines. A minimum length threshold of 30 mm was used, whereas the maximum threshold varied between 90 and 200 mm depending on the tract.

Following fiber reconstruction, filtering procedures were applied to remove noisy streamlines and false positives while preserving, as much as possible, fibers following the anatomical trajectories described for each tract. To refine the initial fiber reconstructions and ensure anatomical plausibility, a multi-step filtering protocol was implemented. Following the generation of the initial 4,000 streamlines, the “Delete Repeated Tracts” function in DSI Studio was applied to identify and eliminate redundant spatial trajectories. This step prevents the artificial inflation of fiber counts and ensures that the resulting bundles represent distinct anatomical connections rather than an overrepresentation.

Subsequently, a “Tract Cleaning” procedure was performed to remove fibers with erratic trajectories or geometric shapes that deviated significantly from the main morphology of the bundle. This individualized approach aimed to identify the most structurally stable and morphologically consistent patterns across the cohort, providing a robust and reliable basis for the subsequent comparative analysis of the twelve investigated pathways.

#### 2.2.5 Atlas tract reconstruction

**Fig. 1:**
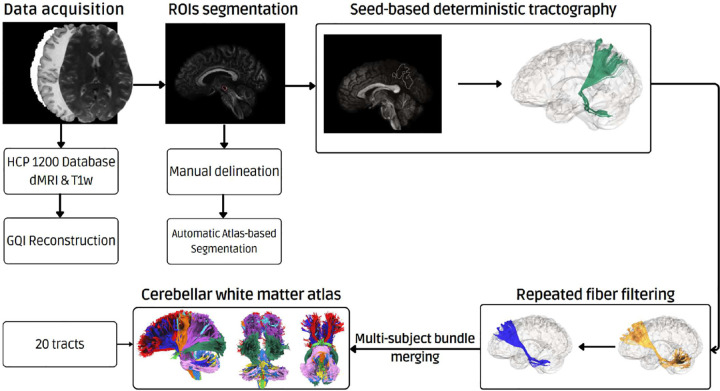
Methodology used to obtain the cerebellar connections atlas.

The final phase of atlas generation involved the fusion of the reconstructed tracts across subjects. First, the tracts from each subject were spatially normalized to MNI space [75] using MRtrix3 [76], by applying the individual nonlinear transformation matrices provided by the HCP database. Subsequently, for each tract, the 20 representative bundles were combined to generate the final multi-subject atlas of cerebellar connections (Fig. 1).

### 2.3 Atlas evaluation and reproducibility analysis

To ensure the reliability and applicability of the atlas, a validation framework was implemented, encompassing both anatomical expert validation and computational evaluation.

#### 2.3.1 Neuroanatomical expert validation

To evaluate structural accuracy, the resulting multi-subject atlas tracts were qualitatively evaluated by a senior neurosurgeon with expertise in neuroanatomy and tractography. The assessment employed a standardized rubric to evaluate four key criteria: (1) connectivity between anatomical regions, (2) trajectory between regions of interest (ROIs), (3) segmented ROIs used for reconstruction, and (4) anatomical coherence along the pathway. Each parameter was scored on a 5-point scale. The evaluator used Supplementary File 1 Section 1, containing the detailed anatomical description of each tract, as a guide for the evaluation.

#### 2.3.2 Automatic bundle segmentation

To automatically segment the atlas tracts in new subjects without human intervention and assess their reproducibility, the segmentation method proposed by Guevara et al. [25] was applied. This method uses a multi-subject atlas as a reference to classify the streamlines from the whole-brain tractography data of a new subject. As a prerequisite, both the atlas fibers and the subject’s streamlines must be normalized to a common spatial reference and resampled to a fixed number of equidistant points, both requirements being necessary to compute meaningful distances between corresponding points. Each streamline is then assigned to the bundle whose centroid yields the smallest distance, provided it falls below a bundle-specific threshold defined a priori. The distance metric used is the normalized maximum Euclidean distance *d*_*NE*_(*S,C*) = *d*_*ME*_(*S,C*) + *NT*, where *d*_*ME*_ (Equation 1) is the maximum Euclidean distance between corresponding points of the streamline *S* and the centroid *C*, accounting for both orientations, and *NT* (Equation 2) penalizes length differences between the streamline *l*_*s*_ and the centroid *l*_*c*_.


(1) $$d_{\text{ME}}(S,C)=\text{min}\left(\underset{k}{\text{max}}\left\|s_{k}-c_{k}\right\|,\underset{k}{\text{max}}\left\|s_{k}-c_{n-k}\right\|\right)$$

(2) $$\text{NT}={\left(\frac{\left|l_{S}-l_{C}\right|}{\text{max}\left(l_{S},l_{C}\right)}+1\right)}^{2}-1$$

To reduce the computational cost of evaluating *d*_*NE*_ over massive fiber datasets, Vázques et al. [77] proposed a progressive discarding algorithm operating in three stages, using 1, 2, and 4 fiber points respectively, reducing segmentation time from hours to minutes without introducing false negatives. Subsequently, Vázquez et al. [78] optimized its spatial complexity from 𝒪(*NM*) to 𝒪(*N* +*M*), enabling the entire fiber set to be loaded into memory, maximizing parallelization and inferring the orientation of each fiber during the process, thereby halving the number of required comparisons. Prior to the automatic bundle segmentation, all normalized tracts from the atlas were resampled to 21 equidistant points, a value selected as sufficiently representative of fiber geometry while minimizing additional computational cost.

#### 2.3.3 Optimal threshold selection

**Fig. 2:**
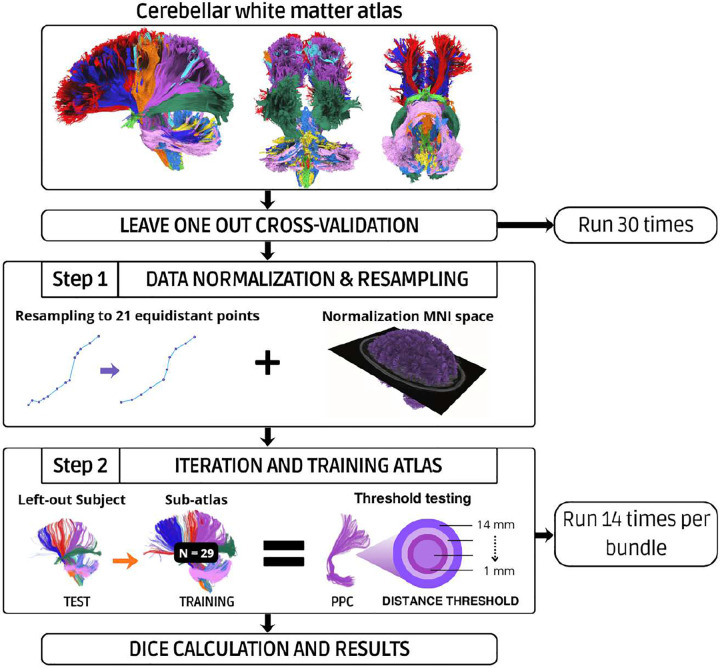
Leave-one-out cross-validation workflow for atlas validation. For each subject, fibers were excluded from the atlas to prevent segmentation bias (Step 1), data were normalized (Step 2), and optimal thresholds were determined by comparing manually segmented tracts with automatically generated tracts (Step 3).

First, we determined the optimal segmentation distance threshold for each atlas tract to enable automatic bundle identification from whole-brain tractography data. For that, we used the atlas tracts generated through interactive manual segmentation to implement a leave-one-out cross-validation strategy (Fig. 2). To this end, 30 versions of the atlas were constructed, each excluding the fibers from a different subject. Thus, each sub-atlas represented the collective information from the remaining 29 subjects, ensuring that the evaluated subject did not contribute to its construction.

Whole-brain deterministic GQI tractography data were generated for each of the 30 HCP subjects included in atlas construction using DSI Studio, with the same parameters employed during tract reconstruction: an angular threshold of 0, corresponding to a randomized range between 45° and 90°, and a total of 3 million streamlines per subject. The tracking threshold was varied across three values (0.04, 0.08, and 0.12), resulting in three tractography datasets with 3 million streamlines per subject. This approach was intended to cover the range of parameters used during the interactive manual construction of the atlas tracts, thereby evaluating the performance of automatic segmentation across different tractography protocols. Each dataset was subsequently transformed to MNI space using the subject-specific transformation matrix and resampled to 21 equidistant points.

Then, for each subject, the whole-brain tractography data were automatically segmented using the corresponding cerebellar connection sub-atlas. Segmentation was initialized with a distance threshold of 15 mm for each of the 20 fascicles, a value sufficiently large to account for the morphological variability of long fiber tracts [25].

The quality of each resulting tract was quantitatively assessed in MNI space by comparing its volumetric mask with the corresponding reference tract mask (*ground truth*), derived from the manual segmentation performed prior to atlas construction. The comparison was performed using the Dice coefficient, computed according to Equation 3, where *M*_*mt*_ denotes the mask of the manually segmented tract and *M*_*at*_ the mask of the automatically segmented tract.


(3) $$\text{Dice}\;\text{Coefficient}=\frac{2\times \text{Volume}\left(M_{\text{mt}}\cap M_{\text{at}}\right)}{\text{Volume}\left(M_{\text{mt}}\right)+\text{Volume}\left(M_{\text{at}}\right)}$$

At each iteration, the distance threshold was reduced by 1 mm until reaching a minimum value of 1 mm. The corresponding Dice coefficient was computed and stored for every threshold value. Upon completion of the process for all subjects, the thresholds yielding Dice coefficients greater than 0.5 were identified for each tract. Subsequently, the two most frequently occurring thresholds were selected as the optimal segmentation thresholds for that tract. This process was performed independently for each of the three tractography datasets generated with tracking thresholds of 0.04, 0.08, and 0.12. As a result, two threshold values were selected per tract for each dataset, yielding a total of six candidate threshold values per fascicle. These parameters constitute the tentative segmentation parameters of the atlas, aiming to ensure robustness and transferability to new datasets.

### 2.4 Reproducibility analysis on independent subjects

To evaluate the reproducibility of the atlas in subjects not involved in its construction, an independent validation cohort of 25 subjects from the HCP 1200 database was used. These subjects were distinct from the 30 participants included in atlas generation. For each subject in the evaluation cohort, the 20 cerebellar connection tracts were manually segmented following the same protocol used during atlas construction, described in Section 2.2.3. These manual segmentations constituted the ground-truth reference tracts for validation.

Analogously to the procedure used for the atlas subjects during automatic segmentation, whole-brain deterministic GQI tractography data were generated for each subject in the validation cohort using DSI Studio. The tracking threshold was varied across the same three values used previously (0.04, 0.08, and 0.12), resulting in three tractography datasets per subject. After calculation, the datasets were normalized to MNI space and resampled with 21 equidistant points. Automatic segmentation of the 20 fascicles was then applied to each of the three tractography datasets. For every tract within each dataset, the two optimal distance thresholds previously derived from the leave-one-out analysis were evaluated.

The results were quantitatively evaluated by comparing the volumetric masks of the automatically segmented tracts with the corresponding manual reference segmentations using the Dice coefficient (Equation 3). This external evaluation assessed the ability of the atlas to automatically identify cerebellar fascicles in an independent group of subjects and to determine which tracts exhibited sufficiently robust performance for use in future studies.

## 3 Results

A total of 1,100 individual tracts were successfully manually reconstructed across all 55 individuals used in this study (30 atlas-generation subjects and 25 evaluation subjects), corresponding to the 20 defined cerebellar connections.

### 3.1 Anatomical ROI Segmentation

**Fig. 3:**
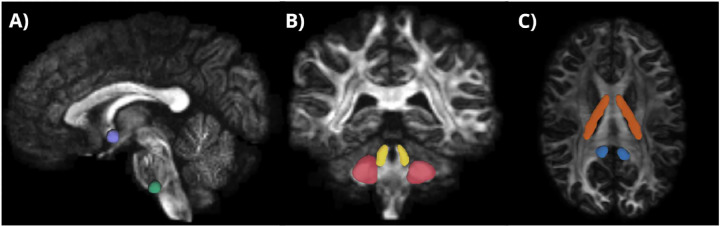
Manual segmentation of the anatomical structures used as regions of interest for tract reconstruction. A) Purple: Hypothalamus; Green: inferior olivary nucleus. B) Yellow: superior cerebellar peduncle (SCP), red: middle cerebellar peduncle. C) Orange: thalamus, blue: inferior cerebellar peduncle (ICP).

**Fig. 4:**
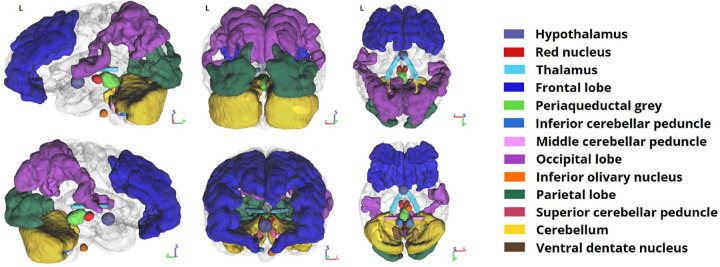
All ROIs used for the reconstruction of cerebellar tracts.

Ten anatomical structures (Table 3) were manually segmented and visually inspected using DSI Studio. Additionally, 13 ROIs (Table 2) were automatically segmented using the built-in atlases available in DSI Studio. The anatomical accuracy of the manually segmented ROIs was visually validated by a specialized medical technologist. As illustrated in Fig. 3, the structures were delineated across three orthogonal planes to optimize boundary visualization. The hypothalamus and the inferior olivary nucleus (ION) were defined in the sagittal plane (Fig. 3A). The middle and superior cerebellar peduncles (MCP and SCP) were delineated in the coronal plane (Fig. 3B), whereas the thalamus and the inferior cerebellar peduncle (ICP) were outlined in the axial plane (Fig. 3C). With the exception of the midline structures (i.e., the ION and the hypothalamus), separate ROIs were generated for the left and right hemispheres of each anatomical structure. Fig. 4 shows the 23 ROIs used for the individual reconstruction of each tract in both the atlas and test subjects.

### 3.2 Atlas tract reconstruction

**Fig. 5:**
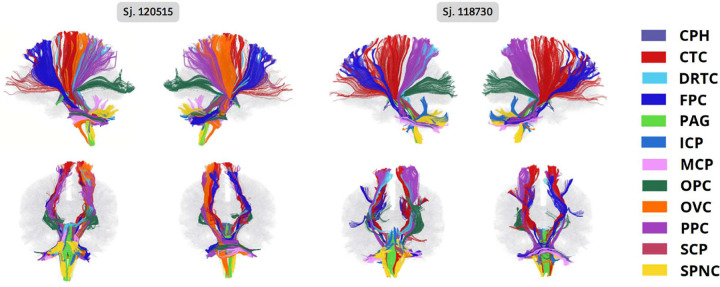
Tract reconstruction for two subjects used in the creation of the cerebellar connection atlas.

The manually and automatically segmented ROIs were used to reconstruct the 600 individual tracts included in the atlas. Subsequently, for all 20 fascicles, the “Delete Repeated Tracts” function in DSI Studio was applied using a minimum distance threshold of 1 mm between voxels to remove redundant fiber. Owing to this restrictive threshold, a substantial number of closely aligned fibers were intentionally preserved to maintain a high bundle density. Manual tract cleaning was then performed to eliminate fibers with trajectories that differed from the anatomical description established for each tract. Fig. 5 illustrates the individual tracts obtained at this final stage for two subjects, prior to merging the tracts from all 20 subjects into single representative bundles for atlas construction.

**Fig. 6:**
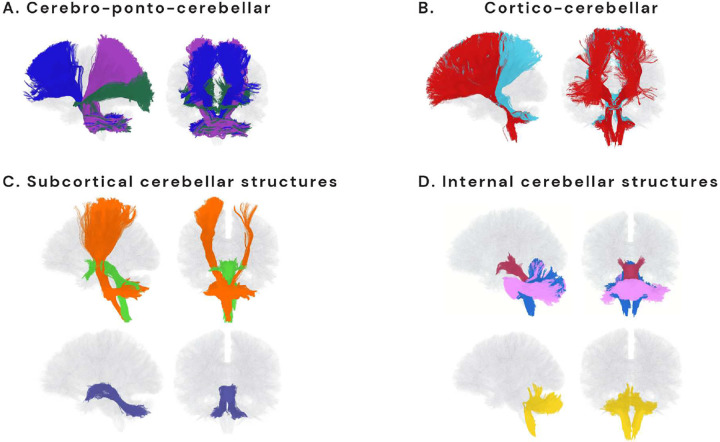
A) Cerebro-ponto-cerebellar tracts included in the atlas. Blue: Fronto-ponto-cerebellar tract (FPC); purple: parieto-ponto-cerebellar tract (PPC); green: occipito-ponto-cerebellar tract (OPC). B) Cortical tracts included in the atlas. red: cerebello-thalamo-cortical (CTC); sky blue: dentato-rubro-thalamo-cortical (DRTC). C) Subcortical tracts included in the atlas. Orange: Olivocerebellar tract (OVC); green: periaqueductal gray cerebellar tract (PAG); light purple: cerebello-ponto-hypothalamic tract (CPH). D) Internal cerebellar tracts. yellow: spinocerebellar tract (SPNC); pink: middle cerebellar peduncle (MCP); rose red: superior cerebellar peduncle (SCP); azure: inferior cerebellar peduncle (ICP).

**Fig. 7:**
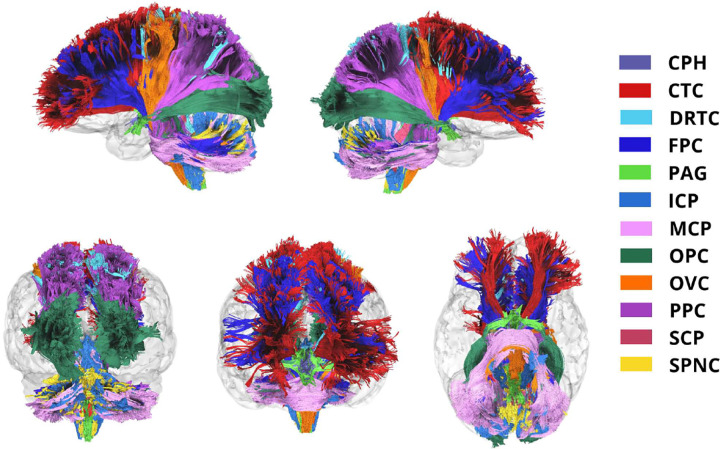
The atlas of cerebellar connections constructed based on 30 subjects from the HCP database.

The cerebro-ponto-cerebellar tracts (Fig. 6A) were reconstructed using the MCP as the seed region and the respective cerebral lobes as target ROIs. The dentato-rubro-thalamo-cortical (DRTC), cerebello-thalamo-cortical (CTC), and cerebello-ponto-hypothalamic (CPH) tracts (Figs. 6B and 6C) were delineated using the ventral dentate nucleus as the seed, with the thalamus, red nucleus, and hypothalamus serving as their respective target ROIs. The spinocerebellar (SPNC) tract was reconstructed using the ICP as the seed and the cerebellar cortex as the ROI. Furthermore, the olivocerebellar (OVC) and periaqueductal gray cerebellar (PAG) tracts were defined using the inferior olivary nucleus (ION) and the periaqueductal gray as their respective target regions (Fig. 6D). Fig. 1 of the Supplementary File 1 presents each tract included in the atlas in a separate subfigure. Finally, the complete multi-subject atlas containing all 20 cerebellar pathways is presented in Fig. 7.

Before publication, a link to the repository where the atlas will be publicly available will be included here.

### 3.3 Neuroanatomical expert validation

A senior neurosurgeon with expertise in neuroanatomy and tractography qualitatively evaluated the 23 segmented ROIs and the 20 atlas tracts. For the evaluation, a detailed anatomical description of the involved ROIs and the trajectories of all atlas tracts was provided, including state-of-the-art references and illustrative figures (see Supplementary File 1 Section 1). The expert was asked to score four criteria using a five-level scale, where a score of 1 indicated that the criterion was not achieved and a score of 5 indicated that it was fully achieved.

1. The **connectivity between anatomical regions** criterion evaluated the correspondence between the reconstructed tract endpoints and the anatomical regions described in the literature. Higher scores were assigned to tracts showing clear and accurate connectivity between the expected origin and termination regions, whereas lower scores reflected partial or no correspondence with these regions.
2. The **trajectory between regions of interest** criterion evaluated the anatomical correspondence of the reconstructed tract pathway with descriptions reported in the literature. Higher scores were assigned to tracts showing trajectories consistent with the expected anatomical course and direction, whereas lower scores reflected increasing spatial deviations, structural inconsistencies, or trajectories unrelated to the known anatomical pathway.
3. The **segmented ROIs used for reconstruction** criterion evaluated the anatomical accuracy and delineation quality of the ROIs used for tract reconstruction. Higher scores were assigned to precisely segmented ROIs that closely adhered to anatomical boundaries, whereas lower scores reflected imprecise delineation, overlap with adjacent structures, or anatomically incorrect segmentations.
4. The **anatomical coherence along the pathway** criterion evaluated the structural continuity and integrity of the reconstructed tract along its trajectory. Higher scores were assigned to tracts exhibiting continuous morphology and consistent fiber organization between regions, whereas lower scores reflected fragmentation, dispersion outside the expected pathway, or the presence of anatomically inconsistent fibers.

The complete spreadsheet containing the expert evaluation is available in Supplementary File 2. It is important to note that assessing the anatomical accuracy of deep gray matter structures within the brainstem (including nuclei such as the dentate nucleus, red nucleus, and PAG) is inherently challenging due to the limited contrast in T1-weighted images, particularly in averaged brain templates such as the MNI template. Additionally, these structures intrinsically lack clearly defined imaging and anatomical boundaries. Consequently, the highest score that could reasonably be assigned to these ROIs by the evaluator was 4 rather than 5.

Table 4 presents the average scores across the four evaluated criteria for each of the 20 cerebellar connections included in the atlas. The results demonstrate that the majority of the proposed tracts (80%) achieved a high level of anatomical fidelity, with mean scores ranging from 3.8 to 4.5 (overall mean of 3.9). Tracts such as the middle cerebellar peduncle (MCP) and the superior cerebellar peduncle (SCP) showed the best results, with scores of 4.5. These were followed by the SPNC tract (4.4) and the cerebro-ponto-cerebellar tracts (FPC 4.4, OPC 4.4, and PPC 3.9). The expert evaluation confirmed that the region of interest (ROI) placement and the resulting streamline trajectories in these pathways were highly accurate and closely aligned with established neuroanatomical descriptions. Meanwhile, in all other tracts, the main factor contributing to lower scores was the presence of false positive streamlines. Additionally, in some tracts, streamlines did not fully reach the cerebellar cortex, or exhibited deviations from the expected anatomical trajectories in specific segments of the tracts. The lowest score was obtained for the OVC tract, which presented a high number of false positives due to the use of a single ROI for both the right and left olivary nuclei.

### 3.4 Optimal segmentation thresholds

The Dice coefficient was calculated to evaluate the performance of automatic segmentation using the sub-atlases across the 30 subjects included in atlas construction, by comparison with their corresponding manual segmentations. Table 5 reports, for each fascicle, the highest Dice coefficient obtained across the leave-one-out analyses, together with the corresponding mean and standard deviation. The results revealed considerable variability across fascicles. The DRTC tract exhibited the poorest performance across all configurations, with Dice coefficients below 0.25 in both hemispheres. Similarly, the OVC tract showed low agreement with the manual segmentations across the different tractography datasets, whereas the CTC tract demonstrated reduced but progressively increasing similarity with higher tracking-threshold values. In contrast, the ICP and MCP tracts achieved the highest Dice coefficients overall. The FPC, OPC, PPC, and SPNC tracts demonstrated relative stability across configurations, with no substantial variation in segmentation similarity. Finally, the PAG and SCP tracts showed the highest agreement between automatic and manual segmentations, constituting the best-performing fascicles across all evaluated configurations. Regarding the three tracking threshold values used, the results were highly similar across the three datasets for all tracts, with slightly better performance observed for the intermediate value of 0.08.

The six candidate threshold values identified for each tract, corresponding to the highest Dice coefficients obtained across all subjects, are presented in Table 6. These thresholds correspond to the two optimal distance thresholds, presented in ascending order (DT1 and DT2), obtained for each tract and for each whole-brain tractography dataset generated using tracking-threshold values of 0.04, 0.08, and 0.12. In general, similar distance thresholds were observed for each fascicle across the different tracking-threshold configurations, with the larger selected threshold (DT2) typically being 1 mm greater than the smaller threshold (DT1). Additionally, as expected, larger thresholds were identified for longer fascicles (10–13 mm), whereas smaller thresholds were obtained for shorter fascicles (3–6 mm).

**Fig. 8:**
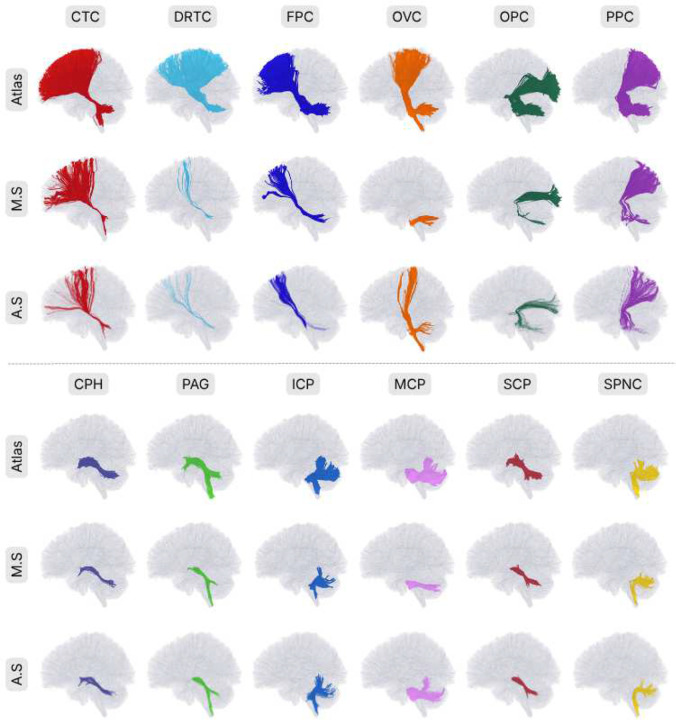
Comparison of cerebellar tract segmentation results for subject ID:117930, a subject included in atlas construction. For each tract, three sagittal views of the left hemisphere are displayed: the atlas reference tract, the manually segmented tract (M.S), and the optimal automatic segmentation (A.S) obtained through the leave-one-out process without human intervention.

Figure 8 qualitatively illustrates the results of the leave-one-out process for a representative atlas subject, displaying three tracts side by side: the atlas tract, the manually segmented tract, and the optimal automatically segmented tract obtained from the leave-one-out analysis. In general, shorter tracts tended to exhibit greater visual similarity across all three representations (CPH, PAG, ICP, MCP, SCP, and SPNC). Conversely, longer tracts such as the FPC, OVC, and OPC showed notable differences in fiber direction and spatial distribution between the manual and automatic segmentations. In the case of the DRTC tract, all three representations contained a reduced number of fibers, hindering direct comparison, which is consistent with the low Dice coefficients reported in Table 5. In contrast, the PAG and SCP tracts showed the highest visual concordance across the three representations, consistent with their superior quantitative performance. Overall, this comparison provides a direct visualization of the degree of agreement between approaches and offers insight into the sources of variability observed in the quantitative analysis.

### 3.5 Atlas validation on a different group

The distance thresholds obtained for the leave-one-out automatic segmentation were subsequently applied to the evaluation group, composed of 25 subjects from the HCP database. The performance of automatic segmentation was also evaluated using the Dice coefficient. Since the second distance threshold (DT2) consistently yielded higher results across all bundles, only the results obtained with this threshold are presented (Table 7). Nonetheless, Fig. 3 of the Supplementary File 1 shows a visual comparison between the 3 tracking thresholds and the two distance thresholds. As observed in the leave-one-out automatic segmentation analysis, the results revealed considerable variability across fascicles, while remaining generally consistent with those obtained for the atlas subjects. The DRTC tract exhibited the poorest overall performance, with mean Dice coefficients at or near zero in several configurations. These findings suggest that this pathway is particularly difficult to identify under the current threshold and tractography parameter settings, although alternative approaches may yield improved results. The OVC and CPH tracts also showed consistently low mean Dice coefficients across all configurations. In contrast, the PAG tract achieved the highest performance, with mean Dice coefficients reaching up to 0.68 and maximum values up to 0.88, indicating that in several subjects this tract was segmented with high spatial agreement relative to the manual reference. Fig. 4 of the Supplementary File 1 shows the automatic segmentation results for the PAG for the 25 subjects. The ICP, MCP, SCP, and SPNC tracts also demonstrated comparatively strong performance. Figs. 5 and 6 in Supplementary File 1 present the results for the three cerebellar peduncles obtained using manual and automatic segmentation, respectively. Notably, although mean Dice coefficients remained moderate across most fascicles, the maximum values observed indicate that several tracts could be automatically segmented with considerable accuracy in a subset of subjects, suggesting that inter-subject anatomical variability plays a substantial role in the overall performance. Fig. 7 of the Supplementary File 1 shows a visual comparison of the manual and automatic segmentation of all the tracts of a test subject, representing different levels of performance. Especially, Fig. 8 of the Supplementary File 1, which shows the lowest Dice coefficient for this specific subject.

## 4 Discussion

The reconstruction of cerebellar connections successfully identified several anatomically consistent pathways, including structures associated with the deep cerebellar nuclei. The prominent involvement of the middle cerebellar peduncle (MCP) is particularly noteworthy due to its major role in cerebro-cerebellar connectivity. Overall, the results reveal variations in tract quality and anatomical consistency across pathways, reflecting both pathway-specific characteristics and inter-subject anatomical variability.

Regarding anatomical connectivity, this work studied the complex structural networks linking the cerebellum and the cerebrum, reconstructing 20 distinct fiber bundles. As illustrated in Fig. 7, these reconstruction results align strongly with established neuroanatomical descriptions documented in previous literature and postmortem studies alongside the included review by the neuroanatomical expert.

These results highlight the complexity of reconstructing cerebellar pathways using tractography, particularly due to the intricate anatomical structures involved. A critical finding of the expert review was the influence of automated ROI segmentation on reconstruction quality. Although the atlas-based automatic segmentations provided a stable framework for pathway reconstruction, the reviewer noted that the cortical and cerebellar ROIs did not always fully encompass the corresponding lobes. Consequently, several “Segmented ROI” categories received a maximum score of only 4. These observations suggest that, while automated tools are efficient for atlas generation, future refinements may benefit from expanded cortical masks to ensure complete lobular coverage. In particular, the thalamic and hypothalamic regions presented substantial challenges, as minor voxel displacements within these small and densely packed nuclei could cause streamlines to deviate toward the interpeduncular cistern or the optic tracts.

Before implementing the automatic segmentation method proposed by Vazquez et al. [78], an appropriate distance threshold had to be determined for each tract. This optimization phase involved the generation of 29 leave-one-out sub-atlases and the evaluation of 14 different threshold values for each of the 20 fascicles. During this iterative process, the optimal threshold for a given tract was found to vary between 3 and 9 mm across subjects. Therefore, the most frequently selected optimal threshold for each fascicle was adopted as the final standardized value. In future work, more advanced segmentation approaches could be explored to improve adaptation to individual anatomical variability.

Quantitative evaluation revealed an average Dice coefficient of 0.33 between the manual and automatic tract masks across the 20 fascicles, with values reaching up to 0.40 depending on the tracking threshold employed. Although the overall spatial overlap was relatively low, specific pathways such as the SCP and PAG tracts achieved higher average Dice scores of up to 0.63 and 0.68, respectively.

The overall modest Dice values may be attributed to the limited sample size used for atlas generation (*N* = 30) and to the inherent difficulty of manually delineating anatomical ROIs. Minor anatomical variations can substantially affect spatial overlap metrics. In addition, the automatically segmented bundles generally contained fewer fibers, significantly influencing the Dice coefficient due to reduced tract coverage. Notably, some tracts could not be automatically segmented in certain subjects, particularly at the highest tracking threshold (*TT*): the DRTC (4 subjects for *TT* = 0.08 and 12 subjects for *TT* = 0.12), the CTC (4 subjects for *TT* = 0.12), and the PPC (3 subjects for *TT* = 0.12) tracts. These isolated failures are likely related to the highly restrictive tracking thresholds, which may have prevented the algorithm from capturing fibers corresponding to the manual segmentations. This interpretation is supported by the slight overall improvement in Dice scores observed when the tracking threshold was set to 0.08.

Nonetheless, a low Dice coefficient does not necessarily indicate an anatomically inaccurate tractography reconstruction, as illustrated in Fig. 8 of Supplementary File 1 (Section 3). Rather, a low Dice value indicates limited spatial overlap between the manual and automatic segmentations, while the automatically segmented bundle may still provide an anatomically plausible representation of the cerebellar pathway. Another important observation, illustrated in Figs. 6 and 7 of Supplementary File 1 (Section 3), concerns the comparison between manual and automatic segmentations of the three cerebellar peduncles. The automatically segmented peduncles exhibited greater consistency across subjects and closer similarity to the atlas representation.

Segmentation, whether manual or automatic, is a fundamental step in mapping structural connectivity through tractography. However, manual delineation is highly time-consuming and prone to inter-subject variability. The development of reliable multi-subject atlases helps mitigate these limitations by enabling rapid, standardized, and reproducible segmentation across diverse cohorts and anatomical structures.

The limitations of this investigation first include the use of deterministic tractography, which is known to be more rigid and less capable of resolving complex fiber configurations and multiple fiber orientations within a voxel, but produces more false positives. Second, the number of subjects used for cerebellar atlas generation was relatively limited; increasing the cohort size would likely improve the representativeness and robustness of the atlas. Furthermore, the manual segmentation of ROIs could be improved; however, achieving higher anatomical precision would require considerable manual effort and expert supervision.

Although several cerebellar atlases have previously been proposed, most are primarily focused on gray matter parcellation rather than on the reconstruction of continuous white matter pathways [33]. Existing atlases incorporating white matter information are generally limited to highly specific cerebellar regions [34] or emphasize brainstem pathways with only partial cerebellar involvement [37]. Consequently, a comprehensive atlas dedicated to the intrinsic cerebellar circuitry and its bidirectional connections with the cerebrum has remained largely unavailable. This gap in the literature is partly explained by the highly complex organization of cerebellar circuitry. Recent advances in diffusion MRI and tractography have progressively enabled the reconstruction of increasingly complex cerebellar pathways, creating new opportunities for the development of standardized anatomical frameworks.

In this context, the multi-subject atlas of cerebellar white matter tracts developed in the present study provides a comprehensive framework for investigating cerebellar structural connectivity. By incorporating 20 reconstructed pathways, including cerebello-ponto-hypothalamic, parieto-ponto-cerebellar, fronto-ponto-cerebellar, occipito-ponto-cerebellar, contralateral cerebello-thalamo-cortical, dentate-rubro-thalamo-cortical, olivocerebellar, dorsal spinocerebellar, and periaqueductal gray–cerebellar, the proposed atlas extends beyond existing cerebellar parcellation approaches and offers a standardized representation of cerebellar fiber anatomy.

This atlas may facilitate future studies exploring the relationship between cerebellar connectivity and both motor and non-motor functions, while also supporting quantitative tractometry analyses, multimodal neuroimaging integration, and investigations of neurological and neuropsychiatric disorders. Furthermore, its standardized anatomical representation may provide valuable support for preoperative neurosurgical planning and future connectomics research.

## 5 Conclusion

In this work, we presented a multi-subject white matter atlas of 20 cerebellar connections reconstructed from diffusion MRI and tractography data from the Human Connectome Project. By providing a standardized representation of cerebellar white matter anatomy from multiple subjects, this atlas addresses an important gap in current neuroanatomical resources, which have traditionally focused primarily on cerebellar gray matter parcellation.

The anatomical validation confirmed that the reconstructed pathways are, in general, morphologically consistent with established white matter anatomy and previously reported neuroanatomical descriptions. In parallel, the computational evaluation demonstrated a consistent reproducibility of the automatic segmentation of these cerebellar tracts in an independent group of subjects. Although some limitations remain, particularly regarding the variability of automatic segmentation and the complexity of small subcortical structures, the proposed framework provides a reliable basis for future methodological improvements and atlas refinement.

Overall, the proposed cerebellar white matter atlas provides a standardized and reproducible framework for the characterization of cerebellar structural connectivity in vivo. By integrating multiple cerebellar pathways within a unified anatomical representation, this work contributes a novel resource for the study of cerebellar organization and connectivity. The proposed atlas also establishes a foundation for future methodological developments in tractography-based cerebellar mapping and large-scale connectivity analyses.

## Supplementary Material

Supplementary File 1 is provided with additional information and figures supporting the results. Supplementary File 2 contains the detailed evaluation performed by the neuroanatomical expert.

Supplementary Files

This is a list of supplementary files associated with this preprint. Click to download.

- SupplementaryFile2.xlsx
- SupplementaryFile1.pdf

## Figures and Tables

**Table 1:** Selected cerebellar connections pathways

Internal cerebellar structures	Cortex cerebellar	Cerebro pontocerebellar	Subcortical structures-cerebellar
Inferior cerebellar peduncle (ICP)	Cerebello-thalamo-cortical (CTC)	Fronto-ponto-cerebellar (FPC)	Cerebello-ponto-hypothalamic (CPH)
Middle cerebellar peduncle (MCP)	Dentato-rubro-thalamo-cortical (DRTC)	Parieto-ponto-cerebellar (PPC)	Olivocerebellar tract (OVC)
Superior cerebellar peduncle (SCP)		Occipito-ponto-cerebellar (OPC)	Periaqueductal gray cerebellar (PAG)
Dorsal spinocerebellar tract (SPNC)			

**Table 2:** Automatically segmented ROIs

	Anatomic Structures	Atlas / Source
	Frontal, Postcentral, Parietal	Campbell [21]
	Lateral Occipital	FreeSurferDKT_Cortical [70]
Left / Right	Red Nucleus	ATAG_basal_ganglia [71]
	Cerebellum	HCP842_tractography [72]
	Ventral Dentate Nucleus	JulichBrain [73]
Midline	PAG (Periaqueductal Gray)	ATAG_basal_ganglia [71]

**Table 3:** Manually segmented ROIs

	Anatomic Structures
Left / Right	ICP, MCP, SCP, Thalamus
Midline	Hypothalamus, Inferior Olivary Nucleus

**Table 4:** Average Evaluation Scores for Reconstructed Tracts Based on External Expert Review

Tract	$\bar{x}$
MCP	4.5
SCP	4.5
SPNC (L/R)	4.4
FPC (L/R)	4.4
OPC (L/R)	4.4
PPC (L/R)	3.9
CPH (L/R)	3.8
PAG	3.8
ICP (L/R)	3.5
DRTC (L/R)	3.3
CTC (L/R)	3.3
OVC	2.8
**Overall Mean**	**3.9**

**Table 5:** Maximum (*max*), mean $\left(\overset{‾}{x}\right)$, and standard deviation (*σ*) of the Dice coefficients associated with the optimal segmentation thresholds for each cerebellar tract automatically segmented across the 30 subjects participating in atlas construction, compared against their corresponding manual segmentations. Results are reported for three tracking threshold values used in whole-brain tractography data generation: 0.04, 0.08, and 0.12. Tract nomenclature is described in Table 1.

	Tracking Threshold 0.04			Tracking Threshold 0.08			Tracking Threshold 0.12		
Tracts	*max*	$\bar{x}$	*σ*	*max*	$\bar{x}$	*σ*	*max*	$\bar{x}$	*σ*
CPH_R	0.63	0.36	0.15	0.70	0.37	0.15	0.58	0.27	0.16
CPH_L	0.64	0.31	0.16	0.63	0.31	0.16	0.66	0.28	0.20
CTC_R	0.69	0.43	0.14	0.69	0.47	0.14	0.59	0.33	0.16
CTC_L	0.61	0.41	0.11	0.58	0.43	0.10	0.56	0.25	0.17
DRTC_R	0.49	0.20	0.13	0.53	0.16	0.16	0.49	0.09	0.13
DRTC_L	0.57	0.23	0.15	0.48	0.18	0.12	0.36	0.12	0.12
FPC_R	0.63	0.47	0.09	0.66	0.49	0.10	0.68	0.48	0.10
FPC_L	0.62	0.46	0.12	0.66	0.47	0.13	0.61	0.45	0.13
ICP_R	0.69	0.53	0.10	0.71	0.56	0.11	0.75	0.58	0.11
ICP_L	0.69	0.50	0.13	0.72	0.52	0.15	0.75	0.54	0.16
MCP	0.66	0.44	0.11	0.69	0.46	0.12	0.75	0.49	0.14
OPC_R	0.50	0.34	0.07	0.50	0.35	0.08	0.59	0.34	0.11
OPC_L	0.47	0.37	0.06	0.49	0.39	0.07	0.58	0.40	0.09
OVC	0.57	0.23	0.16	0.58	0.23	0.16	0.61	0.24	0.17
PAG	0.78	0.62	0.10	0.80	0.69	0.09	0.83	0.69	0.09
PPC_R	0.54	0.43	0.08	0.56	0.44	0.09	0.59	0.45	0.09
PPC_L	0.58	0.39	0.09	0.61	0.42	0.10	0.54	0.37	0.13
SPNC_R	0.66	0.46	0.10	0.67	0.48	0.09	0.66	0.47	0.11
SPNC_L	0.65	0.43	0.14	0.70	0.45	0.14	0.67	0.45	0.14
SCP	0.72	0.51	0.08	0.79	0.63	0.11	0.81	0.60	0.14

**Table 6:** Optimal automatic segmentation distance thresholds (DT1 and DT2) obtained for each tract of the cerebellar connection atlas through leave-one-out cross-validation. For each whole-brain tractography dataset generated using tracking-threshold values of 0.04, 0.08, and 0.12, the two optimal thresholds selected for each bundle are presented.

	Tracking Threshold 0.04		Tracking Threshold 0.08		Tracking Threshold 0.12	
Tracts	DT 1 [mm]	DT 2 [mm]	DT 1 [mm]	DT 2 [mm]	DT 1 [mm]	DT 2 [mm]
CPH_R	3	4	3	4	5	6
CPH_L	4	5	4	5	5	6
CTC_R	7	8	7	8	10	11
CTC_L	7	8	7	8	10	11
DRTC_R	10	11	10	11	12	13
DRTC_L	10	11	10	11	12	13
FPC_R	8	9	8	9	9	10
FPC_L	9	10	9	10	12	13
ICP_R	4	5	3	4	3	4
ICP_L	4	5	3	4	3	4
MCP	4	5	4	5	4	5
OPC_R	12	13	12	13	13	14
OPC_L	12	13	12	13	12	13
OVC	6	7	4	5	5	6
PAG	3	4	2	3	2	3
PPC_R	7	8	8	9	7	8
PPC_L	11	12	11	12	12	13
SPNC_R	5	6	5	6	4	6
SPNC_L	5	6	5	6	4	6
SCP	3	4	2	3	2	3

**Table 7:** Maximum (*max*), mean $\left(\overset{‾}{x}\right)$, and standard deviation (*σ*) of the Dice coefficients obtained for each cerebellar tract automatically segmented across the 25 validation subjects, using the second optimal segmentation distance thresholds (DT 2), which yielded the best overall performance. Results are reported for three whole-brain tractography datasets generated using tracking threshold values of 0.04, 0.08, and 0.12. Tract nomenclature is described in Table 1.

	Tracking Threshold 0.04			Tracking Threshold 0.08			Tracking Threshold 0.12		
Tracts	*max*	$\bar{x}$	*σ*	*max*	$\bar{x}$	*σ*	*max*	$\bar{x}$	*σ*
CPH_R	0.50	0.24	0.15	0.57	0.28	0.17	0.49	0.25	0.25
CPH_L	0.53	0.31	0.14	0.55	0.32	0.15	0.48	0.25	0.25
CTC_R	0.55	0.31	0.16	0.63	0.37	0.17	0.52	0.28	0.28
CTC_L	0.50	0.31	0.16	0.58	0.33	0.18	0.55	0.23	0.23
DRTC_R	0.31	0.16	0.08	0.49	0.19	0.13	0.57	0.09	0.09
DRTC_L	0.34	0.16	0.09	0.50	0.24	0.14	0.46	0.12	0.12
FPC_R	0.58	0.43	0.06	0.60	0.47	0.07	0.58	0.44	0.44
FPC_L	0.58	0.42	0.10	0.62	0.45	0.11	0.60	0.39	0.39
ICP_R	0.70	0.47	0.18	0.72	0.48	0.19	0.67	0.44	0.44
ICP_L	0.69	0.42	0.17	0.72	0.45	0.18	0.66	0.40	0.40
MCP	0.58	0.44	0.08	0.60	0.47	0.11	0.59	0.44	0.44
OPC_R	0.48	0.28	0.11	0.40	0.27	0.09	0.45	0.26	0.26
OPC_L	0.48	0.32	0.13	0.51	0.34	0.15	0.57	0.33	0.33
OVC	0.55	0.32	0.12	0.53	0.32	0.14	0.60	0.33	0.33
PAG	0.71	0.62	0.06	0.88	0.68	0.09	0.83	0.65	0.65
PPC_R	0.54	0.40	0.11	0.53	0.45	0.10	0.55	0.45	0.45
PPC_L	0.58	0.37	0.13	0.63	0.37	0.14	0.55	0.31	0.31
SPNC_R	0.66	0.38	0.18	0.71	0.41	0.18	0.72	0.42	0.42
SPNC_L	0.70	0.43	0.17	0.73	0.47	0.18	0.72	0.46	0.46
SCP	0.66	0.57	0.12	0.78	0.63	0.13	0.81	0.57	0.57

## Data Availability

Before publication, a link to the repository where the atlas will be publicly available will be included here.

## References

[1] Lent R., Azevedo F.A., Andrade-Moraes C.H., Pinto A.V.: How many neurons do you have? some dogmas of quantitative neuroscience under revision. European Journal of Neuroscience 35(1), 1–9 (2012) 22151227 10.1111/j.1460-9568.2011.07923.x

[2] Schmahmann J.D.: The cerebellum and cognition. Neuroscience Letters 688, 62–75 (2019) 29997061 10.1016/j.neulet.2018.07.005

[3] Gatti D., Rinaldi L., Ferreri L., Vecchi T.: The human cerebellum as a hub of the predictive brain. Brain Sciences 11(11), 1492 (2021) 34827491 10.3390/brainsci11111492 PMC8615481

[4] Li H., Yuan Q., Luo Y.-J., Tao W.: A new perspective for understanding the contributions of the cerebellum to reading: The cerebro-cerebellar mapping hypothesis. Neuropsychologia 170, 108231 (2022) 35378104 10.1016/j.neuropsychologia.2022.108231

[5] Mannarelli D., Pauletti C., Missori P., Trompetto C., Cotellessa F., Fattap-posta F., Currà A.: Cerebellum's contribution to attention, executive functions and timing: psychophysiological evidence from event-related potentials. Brain Sciences 13(12), 1683 (2023) 38137131 10.3390/brainsci13121683 PMC10741792

[6] Welniarz Q., Worbe Y., Gallea C.: The forward model: a unifying theory for the role of the cerebellum in motor control and sense of agency. Frontiers in Systems Neuroscience 15, 644059 (2021) 33935660 10.3389/fnsys.2021.644059 PMC8082178

[7] Montgomery J., Bodznick D.: Evolution of the Cerebellar Sense of Self. Oxford University Press, Oxford (2016)

[8] Shin M.A., Park O.T., Shin J.-H.: Anatomical correlates of neuropsychological deficits among patients with the cerebellar stroke. Annals of Rehabilitation Medicine 41(6), 924–934 (2017) 29354568 10.5535/arm.2017.41.6.924 PMC5773435

[9] Beez T., Munoz-Bendix C., Steiger H.-J., Hänggi D.: Functional tracts of the cerebellum—essentials for the neurosurgeon. Neurosurgical Review 44, 273–278 (2021) 32056026 10.1007/s10143-020-01242-1 PMC7851031

[10] Faber J., Kügler D., Bahrami E., Heinz L.-S., Timmann D., Ernst T.M., Deike-Hofmann K., Klockgether T., van de Warrenburg B., van Gaalen J., Reetz K., Romanzetti S., Oz G., Joers J.M., Diedrichsen J., Giunti P., Garcia-Moreno H., Jacobi H., Jende J., de Vries J., Povazan M., Barker P.B., Steiner K.M., Krahe J., Reuter M.: CerebNet: A fast and reliable deep-learning pipeline for detailed cerebellum sub-segmentation. NeuroImage 264, 119703 (2022) 36349595 10.1016/j.neuroimage.2022.119703 PMC9771831

[11] Çavdar S., Esen Aydın A., Algin O., Aydoğmuş E.: Fiber dissection and 3-tesla diffusion tensor tractography of the superior cerebellar peduncle in the human brain: emphasize on the cerebello-hypthalamic fibers. Brain Structure and Function 225, 121–128 (2020) 31776651 10.1007/s00429-019-01985-8

[12] Frontera J.L., Baba Aissa H., Sala R.W., Mailhes-Hamon C., Georgescu I.A., Léna C., Popa D.: Bidirectional control of fear memories by cerebellar neurons projecting to the ventrolateral periaqueductal grey. Nature Communications 11(1), 5207 (2020) 10.1038/s41467-020-18953-0 PMC7566591 33060630

[13] Kang S., Jun S., Baek S.J., Park H., Yamamoto Y., Tanaka-Yamamoto K.: Recent advances in the understanding of specific efferent pathways emerging from the cerebellum. Frontiers in Neuroanatomy 15, 759948 (2021) 34975418 10.3389/fnana.2021.759948 PMC8716603

[14] Romero J.E., Coupé P., Giraud R., Ta V.-T., Fonov V., Park M.T.M., Chakravarty M.M., Voineskos A.N., Manjón J.V.: CERES: A new cerebellum lobule segmentation method. NeuroImage 147, 916–924 (2017) 10.1016/j.neuroimage.2016.11.003 27833012

[15] De Benedictis A., Rossi-Espagnet M.C., Palma L., Carai A., Marras C.E.: Networking of the human cerebellum: From anatomo-functional development to neurosurgical implications. Frontiers in Neurology 13, 806298 (2022) 35185765 10.3389/fneur.2022.806298 PMC8854219

[16] Stoodley C.J., Schmahmann J.D.: Evidence for topographic organization in the cerebellum of motor control versus cognitive and affective processing. Cortex 46(7), 831–844 (2010) 20152963 10.1016/j.cortex.2009.11.008 PMC2873095

[17] Cajal S.R.: Estructura de Los Centros Nerviosos de las Aves (1888). Jiménez y Molina, Madrid (1924)

[18] Ito M.: Cerebellar circuitry as a neuronal machine. Progress in Neurobiology 78(3–5), 272–303 (2006) 16759785 10.1016/j.pneurobio.2006.02.006

[19] Albus J.S.: A theory of cerebellar function. Mathematical Biosciences 10(1), 25–61 (1971) 10.1016/0025-5564(71)90051-4

[20] Tournier J.-D., Mori S., Leemans A.: Diffusion tensor imaging and beyond. Magnetic Resonance in Medicine 65(6), 1532 (2011) 21469191 10.1002/mrm.22924 PMC3366862

[21] Yeh F.-C.: DSI Studio: an integrated tractography platform and fiber data hub for accelerating brain research. Nature Methods 22(8), 1617–1619 (2025) 40707713 10.1038/s41592-025-02762-8 PMC12394933

[22] Alexander A.L., Lee J.E., Lazar M., Field A.S.: Diffusion tensor imaging of the brain. Neurotherapeutics 4(3), 316–329 (2007) 17599699 10.1016/j.nurt.2007.05.011 PMC2041910

[23] Yeh F.-C., Wedeen V.J., Tseng W.-Y.I.: Generalized *q*-sampling imaging. IEEE Transactions on Medical Imaging 29(9), 1626–1635 (2010) 20304721 10.1109/TMI.2010.2045126

[24] Descoteaux M., Deriche R., Knosche T.R., Anwander A.: Deterministic and probabilistic tractography based on complex fibre orientation distributions. IEEE Transactions on Medical Imaging 28(2), 269–286 (2008) 10.1109/TMI.2008.2004424 19188114

[25] Guevara P., Duclap D., Poupon C., Marrakchi-Kacem L., Fillard P., Le Bihan D., Leboyer M., Houenou J., Mangin J.-F.: Automatic fiber bundle segmentation in massive tractography datasets using a multi-subject bundle atlas. NeuroImage 61(4), 1083–1099 (2012) 22414992 10.1016/j.neuroimage.2012.02.071

[26] Zhang F., Wu Y., Norton I., Rigolo L., Rathi Y., Makris N., O'Donnell L.J.: An anatomically curated fiber clustering white matter atlas for consistent white matter tract parcellation across the lifespan. NeuroImage 179, 429–447 (2018) 29920375 10.1016/j.neuroimage.2018.06.027 PMC6080311

[27] Radwan A.M., Sunaert S., Schilling K., Descoteaux M., Landman B.A., Vandenbulcke M., Theys T., Dupont P., Emsell L.: An atlas of white matter anatomy, its variability, and reproducibility based on constrained spherical deconvolution of diffusion MRI. NeuroImage 254, 119029 (2022) 35231632 10.1016/j.neuroimage.2022.119029 PMC10265547

[28] Román C., Hernández C., Figueroa M., Houenou J., Poupon C., Mangin J.-F., Guevara P.: Superficial white matter bundle atlas based on hierarchical fiber clustering over probabilistic tractography data. NeuroImage 262, 119550 (2022) 35944796 10.1016/j.neuroimage.2022.119550

[29] Habas C., Manto M.: Probing the neuroanatomy of the cerebellum using tractography. Handbook of Clinical Neurology 154, 235–249 (2018) 29903442 10.1016/B978-0-444-63956-1.00014-X

[30] Dell'Acqua F., Descoteaux M., Leemans A.: Handbook of Diffusion MR Tractography: Imaging Methods, Biophysical Models, Algorithms and Applications. Academic Press, London (2024)

[31] Argyropoulos G.D., Christidi F., Karavasilis E., Velonakis G., Antoniou A., Bede P., Seimenis I., Kelekis N., Douzenis A., Papakonstantinou O., Efstathopoulos E., Ferentinos P.: Cerebro-cerebellar white matter connectivity in bipolar disorder and associated polarity subphenotypes. Progress in Neuro-Psychopharmacology and Biological Psychiatry 104, 110034 (2021) 32710925 10.1016/j.pnpbp.2020.110034

[32] Diedrichsen J.: A spatially unbiased atlas template of the human cerebellum. NeuroImage 33(1), 127–138 (2006) 16904911 10.1016/j.neuroimage.2006.05.056

[33] Diedrichsen J., Balsters J.H., Flavell J., Cussans E., Ramnani N.: A probabilistic MR atlas of the human cerebellum. NeuroImage 46(1), 39–46 (2009) 19457380 10.1016/j.neuroimage.2009.01.045

[34] Lyu W., Wu Y., Huynh K.M., Ahmad S., Yap P.-T.: A multimodal submillimeter MRI atlas of the human cerebellum. Scientific Reports 14(1), 5622 (2024) 38453991 10.1038/s41598-024-55412-y PMC10920891

[35] Wu Y., Hong Y., Feng Y., Shen D., Yap P.-T.: Mitigating gyral bias in cortical tractography via asymmetric fiber orientation distributions. Medical Image Aalysis 59, 101543 (2020) 10.1016/j.media.2019.101543 PMC6935166 31670139

[36] Wu Y., Hong Y., Ahmad S., Yap P.-T.: Active cortex tractography. In: International Conference on Medical Image Computing and Computer-Assisted Intervention, pp. 467–476 (2021). Springer 10.1007/978-3-030-87234-2_44 PMC9355463 35939282

[37] Tang Y., Sun W., Toga A.W., Ringman J.M., Shi Y.: A probabilistic atlas of human brainstem pathways based on connectome imaging data. NeuroImage 169, 227–239 (2018) 29253653 10.1016/j.neuroimage.2017.12.042 PMC5856609

[38] Project H.C.: HCP young adult, 1200 subjects data release (2017)

[39] Sotiropoulos S.N., Moeller S., Jbabdi S., Xu J., Andersson J.L., Auerbach E.J., Yacoub E., Feinberg D., Setsompop K., Wald L.L., Behrens T.E.J., Ugurbil K., Lenglet C.: Effects of image reconstruction on fiber orientation mapping from multichannel diffusion MRI: reducing the noise floor using SENSE. Frontiers in Medicine 70(6), 1682–1689 (2013) 10.1002/mrm.24623 PMC3657588 23401137

[40] Glasser M.F., Sotiropoulos S.N., Wilson J.A., Coalson T.S., Fischl B., Andersson J.L., Xu J., Jbabdi S., Webster M., Polimeni J.R., Van Essen D.C., Jenkinson M.: The minimal preprocessing pipelines for the human Connectome Project. NeuroImage 80, 105–124 (2013) 23668970 10.1016/j.neuroimage.2013.04.127 PMC3720813

[41] Andersson J.L., Skare S., Ashburner J.: How to correct susceptibility distortions in spin-echo echo-planar images: application to diffusion tensor imaging. NeuroImage 20(2), 870–888 (2003) 14568458 10.1016/S1053-8119(03)00336-7

[42] Andersson J.L., Sotiropoulos S.N.: Non-parametric representation and prediction of single-and multi-shell diffusion-weighted MRI data using Gaussian processes. NeuroImage 122, 166–176 (2015) 26236030 10.1016/j.neuroimage.2015.07.067 PMC4627362

[43] Andersson J., Smith S., Jenkinson M.: FNIRT-FMRIB's non-linear image registration tool. Human Brain Mapping 2008 (2008)

[44] Van Essen D.C., Smith S.M., Barch D.M., Behrens T.E.J., Yacoub E., Ugurbil K.: The WU-Minn human connectome project: an overview. NeuroImage 80, 62–79 (2013) 23684880 10.1016/j.neuroimage.2013.05.041 PMC3724347

[45] Karavasilis E., Christidi F., Velonakis G., Giavri Z., Kelekis N.L., Efstathopoulos E.P., Evdokimidis I., Dellatolas G.: Ipsilateral and contralateral cerebro-cerebellar white matter connections: a diffusion tensor imaging study in healthy adults. Journal of Neuroradiology 46(1), 52–60 (2019) 30098370 10.1016/j.neurad.2018.07.004

[46] Sival D.A., Noort S.A., Tijssen M.A., Koning T.J., Verbeek D.S.: Developmental neurobiology of cerebellar and basal ganglia connections. European Journal of Paediatric Neurology 36, 123–129 (2022) 34954622 10.1016/j.ejpn.2021.12.001

[47] Machado Filho W.S., Martinez A.R.M., Junior M.C.F.: Neurophysiology of the cerebellum and clinical correlations: a review. Arquivos de Neuro-Psiquiatria 83(08), 001–008 (2025) 10.1055/s-0045-1811623 PMC12449109 40973045

[48] Yin H., Zong F., Deng X., Zhang D., Zhang Y., Wang S., Wang Y., Zhao J.: The language-related cerebro-cerebellar pathway in humans: a diffusion imaging–based tractographic study. Quantitative Imaging in Medicine and Surgery 13(3), 1399 (2023) 36915351 10.21037/qims-22-303 PMC10006158

[49] Liu B., Li W., Zhang Y., Yu T., Cui Z., Xu X., Xu J., Chen X., Feng J., Mao Z., Yang J.: Distinct Roles of Cerebellar Afferent and Efferent Fiber Tracts in Craniocervical Dystonia. Movement Disorders 41(2), 384–394 (2026) 41235506 10.1002/mds.70118 PMC12951270

[50] Zhang P., Duan L., Ou Y., Ling Q., Cao L., Qian H., Zhang J., Wang J., Yuan X.: The cerebellum and cognitive neural networks. Frontiers in Human Neuroscience 17, 1197459 (2023) 37576472 10.3389/fnhum.2023.1197459 PMC10416251

[51] Diedrichsen J., McDougle S.D.: How does the cerebellum contribute to cognitive functions? PLOS Biology 24(3), 3003688 (2026) 10.1371/journal.pbio.3003688 PMC13008071 41871032

[52] Nieuwhof F., Toni I., Dirkx M.F., Gallea C., Vidailhet M., Buijink A.W., Rootselaar A.-F., Warrenburg B.P., Helmich R.C.: Cerebello-thalamic activity drives an abnormal motor network into dystonic tremor. NeuroImage: Clinical 33, 102919 (2022) 34929584 10.1016/j.nicl.2021.102919 PMC8688717

[53] Ji Q., McAfee S.S., Scoggins M., Holtrop J., Glass J.O., Yuan X., Liang J., Li Y., Chiang J., Orr B.A., Edwards A., Storment D., Brinkman T., Robinson G.W., Gajjar A., Reddick W.E., Patay Z., Khan R.B., Bag A.K.: Cerebellar mutism syndrome and dentato-thalamo-cortical tract disruption in diffusion tractography following surgery for medulloblastoma. Radiology 311(2), 232521 (2024) 10.1148/radiol.232521 PMC11140529 38742969

[54] Cocozza S., Bosticardo S., Battocchio M., Corben L., Delatycki M., Egan G., Georgiou-Karistianis N., Monti S., Palma G., Pane C., Saccà F., Schiavi S., Selvadurai L., Tranfa M., Daducci A., Brunetti A., Harding I.H.: Gradient of microstructural damage along the dentato-thalamo-cortical tract in Friedreich ataxia. Annals of Clinical and Translational Neurology 11(7), 1691–1702 (2024) 38952134 10.1002/acn3.52048 PMC11251475

[55] Parlatini V., Itahashi T., Lee Y., Liu S., Nguyen T.T., Aoki Y.Y., Forkel S.J., Catani M., Rubia K., Zhou J.H., Murphy D.G., Cortese S.: White matter alterations in Attention-Deficit/Hyperactivity Disorder (ADHD): a systematic review of 129 diffusion imaging studies with meta-analysis. Molecular Psychiatry 28(10), 4098–4123 (2023) 37479785 10.1038/s41380-023-02173-1 PMC10827669

[56] Parkkinen S., Radua J., Andrews D.S., Murphy D., Dell'Acqua F., Parlatini V.: Cerebellar network alterations in adult attention-deficit/hyperactivity disorder. Journal of Psychiatry and Neuroscience 49(4), 233–241 (2024) 10.1503/jpn.230146 PMC11230668 38960626

[57] Baran O., Baydin S., Mirkhasilova M., Bayramli N., Bilgin B., Middlebrooks E., Ozlen F., Tanriover N.: Microsurgical anatomy and surgical exposure of the cerebellar peduncles. Neurosurgical Review, 1–23 (2022) 10.1007/s10143-021-01701-3 34997381

[58] Keser Z., Hasan K.M., Mwangi B.I., Kamali A., Ucisik-Keser F.E., Riascos R.F., Yozbatiran N., Francisco G.E., Narayana P.A.: Diffusion tensor imaging of the human cerebellar pathways and their interplay with cerebral macrostructure. Frontiers in Neuroanatomy 9, 41 (2015) 25904851 10.3389/fnana.2015.00041 PMC4389543

[59] Kamali A., Kramer L.A., Frye R.E., Butler I.J., Hasan K.M.: Diffusion tensor tractography of the human brain cortico-ponto-cerebellar pathways: a quantitative preliminary study. Journal of Frontiers Imaging 32(4), 809–817 (2010) 10.1002/jmri.22330 PMC4492525 20882611

[60] Sugihara I., Wu H.-S., Shinoda Y.: Morphology of single olivocerebellar axons labeled with biotinylated dextran amine in the rat. Journal of Comparative Neurology 414(2), 131–148 (1999) 10516588

[61] Luo Y., Sugihara I.: The olivocerebellar tract. In: Essentials of Cerebellum and Cerebellar Disorders: A Primer For Graduate Students, pp. 41–45. Springer, Cham (2023)

[62] Granziera C., Schmahmann J.D., Hadjikhani N., Meyer H., Meuli R., Wedeen V., Krueger G.: Diffusion spectrum imaging shows the structural basis of functional cerebellar circuits in the human cerebellum in vivo. PLOS One 4(4), 5101 (2009) 10.1371/journal.pone.0005101 PMC2659746 19340289

[63] Kamali A., Karbasian N., Rabiei P., Cano A., Riascos R.F., Tandon N., Arevalo O., Ocasio L., Younes K., Khayat-khoei M., Mirbagheri S., Hasan K.M.: Revealing the cerebello-ponto-hypothalamic pathway in the human brain. Neuroscience Letters 677, 1–5 (2018) 29673951 10.1016/j.neulet.2018.04.024

[64] Zanchetti A., Zoccolini A.: Autonomic hypothalamic outbursts elicited by cerebellar stimulation. Journal of Neurophysiology 17(5), 475–483 (1954) 13201979 10.1152/jn.1954.17.5.475

[65] Park S.Y., Yeo S.S., Jang S.H., Cho I.H.: Anatomical location of the vestibulocerebellar tract in the healthy human brain: a diffusion tensor imaging study. Brain Sciences 11(2), 199 (2021) 33562805 10.3390/brainsci11020199 PMC7914725

[66] Yeo S.S., Park S.Y., Cho I.H.: Injury of the vestibulocerebellar tract in a patient with intracerebral hemorrhage: A case report. Neuroscience Letters 783, 136723 (2022) 35691437 10.1016/j.neulet.2022.136723

[67] Palesi F., Tournier J.-D., Calamante F., Muhlert N., Castellazzi G., Chard D., D'Angelo E., Wheeler-Kingshott C.A.: Contralateral cerebello-thalamo-cortical pathways with prominent involvement of associative areas in humans in vivo. Brain Structure and Function 220(6), 3369–3384 (2015) 25134682 10.1007/s00429-014-0861-2 PMC4575696

[68] Palesi F., De Rinaldis A., Castellazzi G., Calamante F., Muhlert N., Chard D., Tournier J.D., Magenes G., D'Angelo E., Gandini Wheeler-Kingshott C.A.: Contralateral cortico-ponto-cerebellar pathways reconstruction in humans in vivo: implications for reciprocal cerebro-cerebellar structural connectivity in motor and non-motor areas. Scientific Reports 7(1), 12841 (2017) 28993670 10.1038/s41598-017-13079-8 PMC5634467

[69] Cacciola A., Bertino S., Basile G.A., Di Mauro D., Calamuneri A., Chillemi G., Duca A., Bruschetta D., Flace P., Favaloro A., Calabrò R.S., Anastasi G., Milardi D.: Mapping the structural connectivity between the periaqueductal gray and the cerebellum in humans. Brain Structure and Function 224(6), 2153–2165 (2019) 31165919 10.1007/s00429-019-01893-x PMC6591182

[70] Fischl B.: Freesurfer. NeuroImage 62(2), 774–781 (2012) 22248573 10.1016/j.neuroimage.2012.01.021 PMC3685476

[71] Keuken M.C., Bazin P.-L., Crown L., Hootsmans J., Laufer A., Müller-Axt C., Sier R., van der Putten E.J., Schäfer A., Turner R., Forstmann B.U.: Quantifying inter-individual anatomical variability in the subcortex using 7 T structural MRI. NeuroImage 94, 40–46 (2014) 24650599 10.1016/j.neuroimage.2014.03.032

[72] Yeh F.-C., Panesar S., Fernandes D., Meola A., Yoshino M., Fernandez-Miranda J.C., Vettel J.M., Verstynen T.: Population-averaged atlas of the macroscale human structural connectome and its network topology. NeuroImage 178, 57–68 (2018) 29758339 10.1016/j.neuroimage.2018.05.027 PMC6921501

[73] Amunts K., Mohlberg H., Bludau S., Zilles K.: Julich-brain: A 3D probabilistic atlas of the human brain's cytoarchitecture. Science 369(6506), 988–992 (2020) 32732281 10.1126/science.abb4588

[74] Yeh F.-C., Verstynen T.D., Wang Y., Fernández-Miranda J.C., Tseng W.-Y.I.: Deterministic diffusion fiber tracking improved by quantitative anisotropy. PLOS One 8(11), 80713 (2013) 10.1371/journal.pone.0080713 PMC3858183 24348913

[75] Fonov V., Evans A.C., Botteron K., Almli C.R., McKinstry R.C., Collins D.L., Brain Development Cooperative Group: Unbiased average age-appropriate atlases for pediatric studies. NeuroImage 54(1), 313–327 (2011) 20656036 10.1016/j.neuroimage.2010.07.033 PMC2962759

[76] Tournier J.-D., Smith R., Raffelt D., Tabbara R., Dhollander T., Pietsch M., Christiaens D., Jeurissen B., Yeh C.-H., Connelly A.: MRtrix3: A fast, flexible and open software framework for medical image processing and visualisation. NeuroImage 202(116137), 116137 (2019) 31473352 10.1016/j.neuroimage.2019.116137

[77] Vázquez A., López-López N., Sánchez A., Houenou J., Poupon C., Mangin J.-F., Hernández C., Guevara P.: FFclust: Fast fiber clustering for large tractography datasets for a detailed study of brain connectivity. NeuroImage 220, 117070 (2020) 32599269 10.1016/j.neuroimage.2020.117070

[78] Vázquez A., López-López N., Labra N., Figueroa M., Poupon C., Mangin J.-F., Hernández C., Guevara P.: Parallel optimization of fiber bundle segmentation for massive tractography datasets, 178–181 (2019). IEEE

